# Global transcriptome changes of elongating internode of sugarcane in response to mepiquat chloride

**DOI:** 10.1186/s12864-020-07352-w

**Published:** 2021-01-25

**Authors:** Rongfa Chen, Yegeng Fan, Huiwen Zhou, Shanping Mo, Zhongfeng Zhou, Haifeng Yan, Ting Luo, Xing Huang, Mengling Weng, Prakash Lakshmanan, Yangrui Li, Lihang Qiu, Jianming Wu

**Affiliations:** Sugarcane Research Institute, Guangxi Academy of Agricultural Sciences/Sugarcane Research Center, Chinese Academy of Agricultural Sciences, No. 172, East Daxue Road, Nanning, 530007 Guangxi China

**Keywords:** Mepiquat chloride, Sugarcane, Full-length transcriptome, RNA-seq, Growth, Internode

## Abstract

**Background:**

Mepiquat chloride (DPC) is a chemical that is extensively used to control internode growth and create compact canopies in cultured plants. Previous studies have suggested that DPC could also inhibit gibberellin biosynthesis in sugarcane. Unfortunately, the molecular mechanism underlying the suppressive effects of DPC on plant growth is still largely unknown.

**Results:**

In the present study, we first obtained high-quality long transcripts from the internodes of sugarcane using the PacBio Sequel System. A total of 72,671 isoforms, with N50 at 3073, were generated. These long isoforms were used as a reference for the subsequent RNA-seq. Afterwards, short reads generated from the Illumina HiSeq 4000 platform were used to compare the differentially expressed genes in both the DPC and the control groups. Transcriptome profiling showed that most significant gene changes occurred after six days post DPC treatment. These genes were related to plant hormone signal transduction and biosynthesis of several metabolites, indicating that DPC affected multiple pathways, in addition to suppressing gibberellin biosynthesis. The network of DPC on the key stage was illustrated by weighted gene co-expression network analysis (WGCNA). Among the 36 constructed modules, the top positive correlated module, at the stage of six days post spraying DPC, was sienna3. Notably, Stf0 sulfotransferase, cyclin-like F-box, and HOX12 were the hub genes in sienna3 that had high correlation with other genes in this module. Furthermore, the qPCR validated the high accuracy of the RNA-seq results.

**Conclusion:**

Taken together, we have demonstrated the key role of these genes in DPC-induced growth inhibition in sugarcane.

**Supplementary Information:**

The online version contains supplementary material available at 10.1186/s12864-020-07352-w.

## Background

Hormone regulation in plant culturing has been widely used to control the quality of agricultural and horticultural products [1]. Several hormones are known to affect the regulation and co-ordination of plant growth [2]. To date, auxins [3], gibberellins (GA) [4], cytokinins (CTK) [5], abscisic acid (ABA) [6], ethyne (ETH) [7], and brassinosteroids (BR) [8] have been the most popular hormones for stimulating growth in crops. However, growth performance is not the only parameter that is sought after in the increasing demands made by farmers. For example, with excessive vegetative growth, crops such as cotton and sugarcane can hardly be controlled leading to height irregularities in farmland, which results in low productivity [9, 10]. Thus, other regulated chemicals have been introduced as alternatives to inhibit the relevant hormonal pathways.

Mepiquat chloride (DPC) is a well-known chemical that controls organism growth by suppressing the GA pathways [11, 12]. As an exogenous plant growth regulator, DPC is a water-soluble substance that can be applied via spraying in farmlands [13]. With low-dose DPC treatment, studies have seen reduced internode elongation and plant height [13, 14]. Additionally, recent studies have revealed that DPC could also regulate the synthesis of endogenous hormones, carbohydrates, enzymes, and other organic molecules [15, 16]. DPC treatment increased concentrations of chlorophyll, free proline, and soluble proteins, but depressed malondialdehyde levels, contributing to improved resistance to stress [17–19]. In addition, DPC promoted the increase of calcium and phosphorus levels in leaves to strengthen their ability to resist disease [20, 21]. Theoretically, it does this by regulating CTKs and the synthesis of GAs, as well as controlling the ratios of CTKs:GAs- and DPC-mediated rhizogenesis [22]. However, the function and regulatory role of DPC is far from being systematically understood.

Sugarcane is a major agricultural crop for sugar production worldwide [23–25]. About 80% of the world’s sugar is isolated from sugarcane, making it a critical bioenergy crop [26]. Sucrose is primarily generated in the crop’s stem and higher shoot [27, 28], and the internode elongation of stems is associated with the deposition of sucrose [29]. In this situation, GA is employed to stimulate internode elongation [30]. However, rapid stem growth may lead to lower sucrose accumulation [31, 32]. Therefore, how to achieve an ideal balance for the most productive rate of stem growth is the key question in sugar production. In an attempt at solving this problem, DPC was introduced to control the negative effects of GA treatment [33]. Although DPC is widely recognized as a regulator of GA and promotes resistance to stress [34, 35], its underlying molecular mechanism is still unknown. Moreover, to venture into this knowledge would require thorough scanning of the systematic regulation of DPC in plants.

A previous study showed that during internode elongation, regulation by the microRNA-mRNA network in zeatin biosynthesis, nitrogen metabolism, and plant hormone signal transduction pathways played a part in stem growth in sugarcane [36, 37]. These effects may be mediated by GA20-oxidase (GA20-OX1) and a gibberellin receptor (GID1). DPC has shown inhibitory effects on GA generation by suppressing the activities of copalyl diphosphate synthase and *ent*-kaurene synthase [13]. These results revealed the molecular mechanism in controlling growth performance by DPC. However, a vast amount of information about the roles of DPC in growth and resistance to stress remains unknown. Herein, we used the mathematical method, weighted gene co-expression network analysis (WGCNA), to identify key gene networks and hub genes [38–40]. The present study focused on the transcriptome changes induced by DPC treatment using the Illumina HiSeq 4000 platform. The evidence presented here provides new insights on DPC function in controlling stem growth as well as regulating resistance to stress, which are the two most economically important traits in sugarcane.

## Results

### Growth performance

**Fig. 1:**
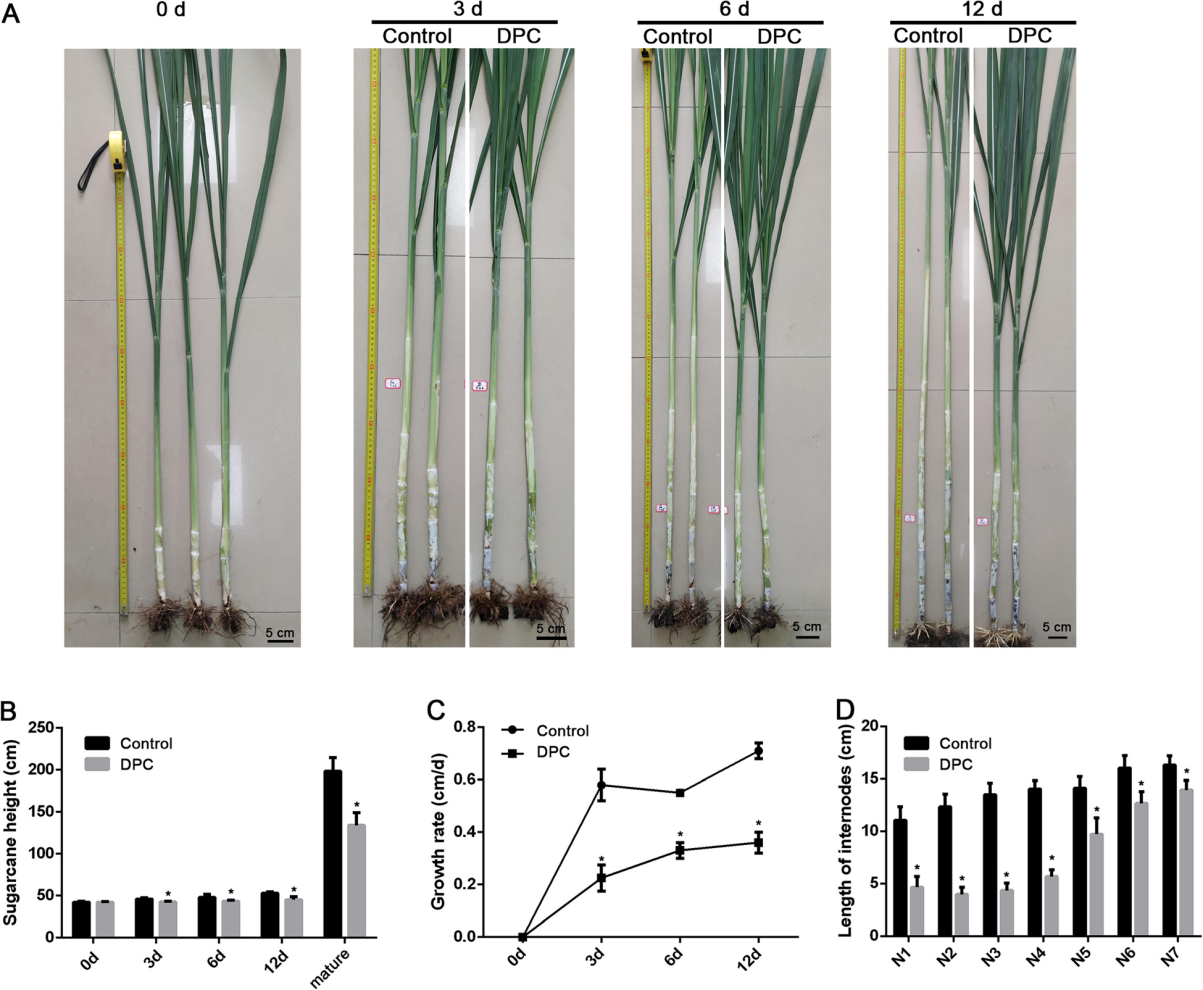
Effects of DPC on sugarcane growth performance on different days after treatment. **a** The growth performance of sugarcane in 0, 3, 6, and 12 days from control and DPC groups. **b** The height of sugarcane on different days after DPC treatment (*n* = 4). **c** The growth rate of sugarcane on different days after DPC treatment (*n* = 4; mature period, *n* = 10). **d** The internode length of sugarcane in mature sugarcane after DPC treatment. * indicates *P*< 0.05

The growth performance of each group at different days were shown in Fig. 1a. At the beginning of the experiment (0 days), no significant difference was found between the control and DPC groups (*P*> 0.05). However, the sugarcane heights on days 3, 6, and 12 as well as that of mature sugarcane, were significantly higher in the control than in the DPC groups (*P*< 0.05) (Fig. 1b). Contrary to the sugarcane height, the growth rates of DPC groups were significantly lower on days 3, 6, and 12 when compared to the control (*P*< 0.05) (Fig. 1c). Moreover, all the internodes were significantly longer in the control group (Fig. 1d).

### Full-length transcriptome of sugarcane

To generate a high-accuracy reference for read mapping data, full-length mRNA sequencing was performed using the PacBio Sequel platform on internodes from mature sugarcane. A total of 17 billion raw reads were obtained. The average length was 2718 bp and N50 was 3011 bp. After circular-consensus sequence (CCS) extraction, 428,444 reads were identified. Among these reads, 348,840 (81.42%) were full-length reads containing 5′ adaptors, poly(A) tail signals, and 3′ adaptors. Meanwhile, 999 million full-length non-chimeric (FLNC) reads with an average length of 2906 bp were identified. These FLNC reads from the cDNA library contain repetitive isoforms that provide data for analysis of isoforms by alignment and assignment to different clusters. The present full-length transcriptome generated 72,671 isoforms. Of these, the average length was 2888.94 bp and the N50 was 3073 (Additional file 2).

**Fig. 2:**
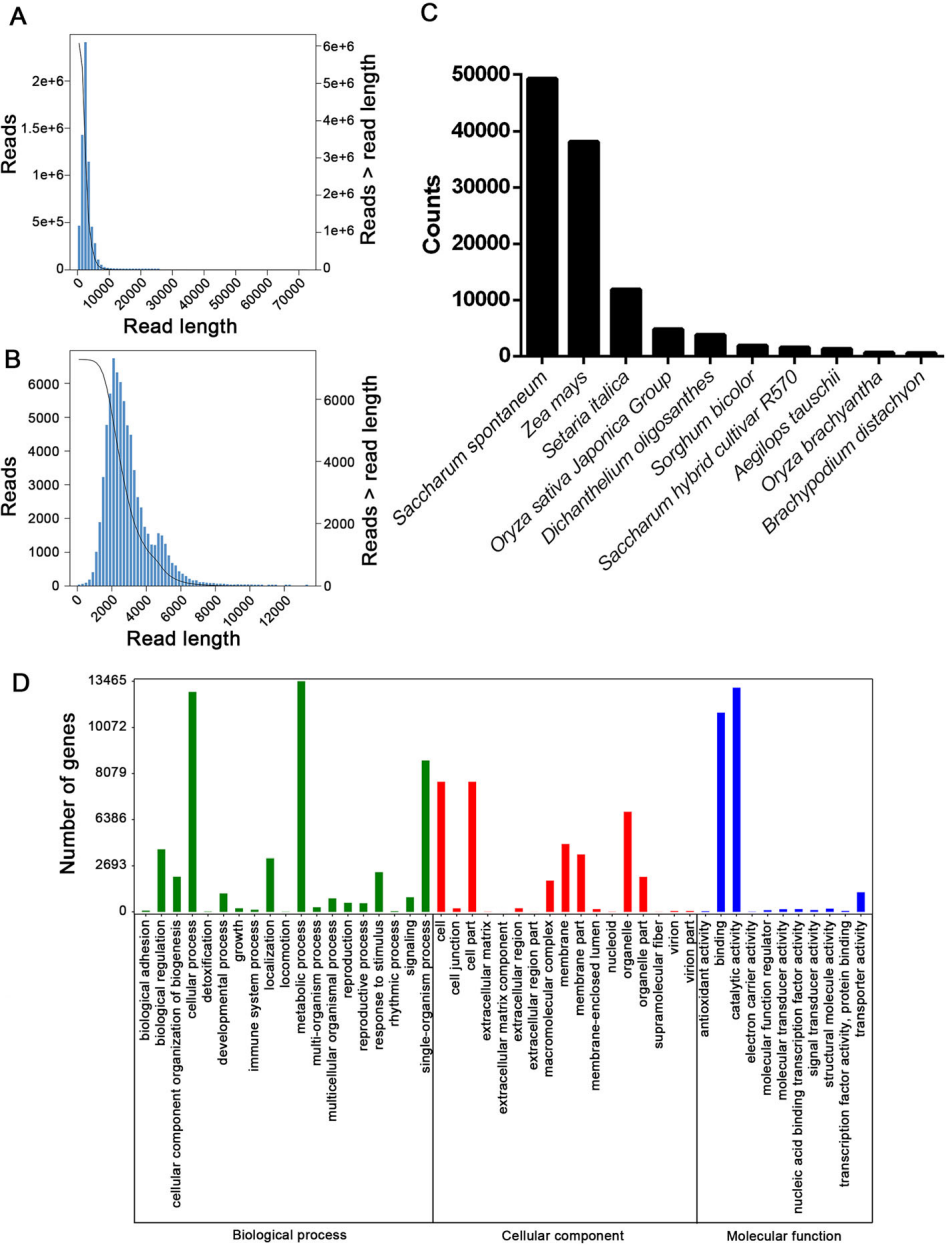
Full-length transcriptome of internode of sugarcane. **a** Length distribution of reads generated from PacBio Sequel System sequencing. **b** Length distribution of isoforms generated from PacBio Sequel System sequencing. **c** Distribution of annotated genes from nr database in different species. **d** GO annotation of the isoforms

The isoforms were annotated by aligning the protein and nucleotide databases. In total 69,803, 56,843, 47,438, and 30,240 isoforms were annotated from nr, Swissport, KOG, and KEGG, respectively. Combining these results, a total of 69,867 isoforms were annotated (Additional file 3). The isoforms were also aligned to different species. The five species with the most hit sequences were *Saccharum spontaneum*, *Setaria italica*, the *Oryza sativa Japonica* group, *Dichanthelium oligosanthes*, and *Sorghum bicolor*. In addition to this, these isoforms were annotated by GO terms assigned to three categories: biological process (50,805 isoforms), cellular component (32,922 isoforms), and molecular function (26,696 isoforms). In the biological process category, metabolic process (13,462 isoforms) and cellular process (12,836 isoforms) were the two most functional terms. Cell (7598 isoforms) and cell parts (7597 isoforms) were the two most functional terms in the cellular component category, while in the molecular function category, catalytic activity (13,086 isoforms) and binding (11,642 isoforms) were the two most functional terms (Fig. 2c).

### DEGs by DPC treatment

**Fig. 3:**
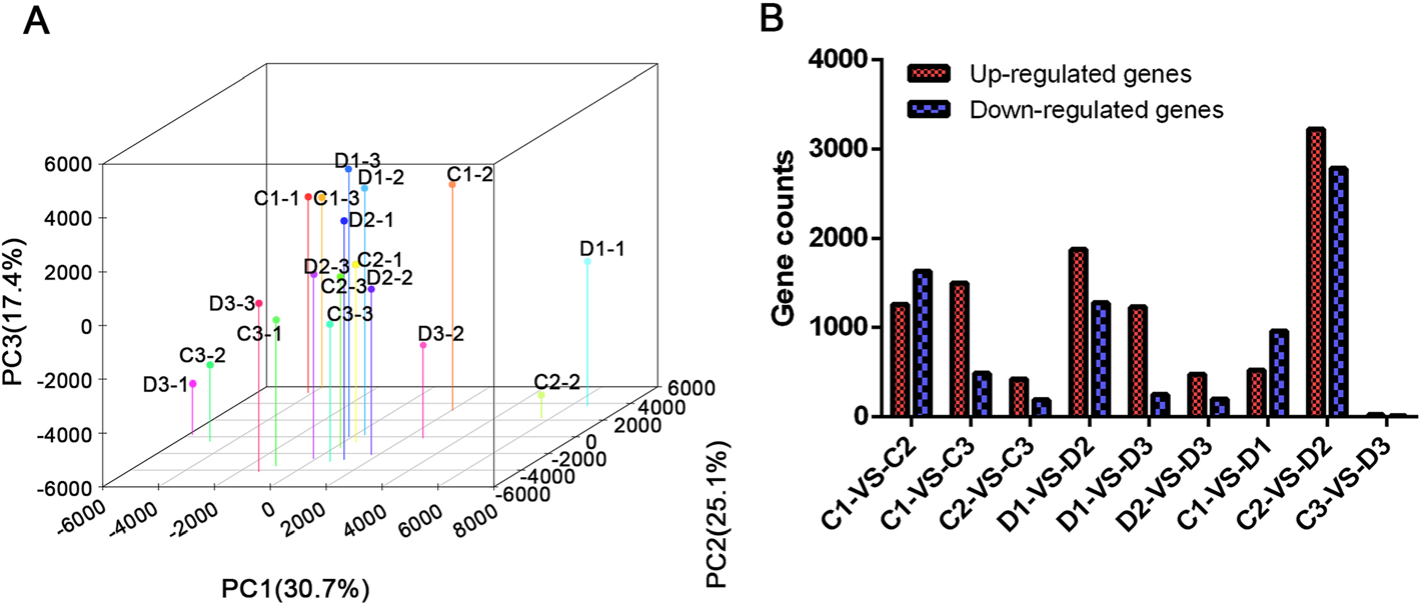
Expression profile analysis based on RNA-seq result. **a** Principle component analyses of the 18 transcriptomes from the internodes of sugarcane on different days, in the control and DPC treatment groups, based on the FPKM. **b** Number of upregulated and downregulated genes of pairwise comparisons

The 150 pair-end reads were obtained for DEG analysis. In total, 1,404,530,300 raw reads were generated from 18 cDNA libraries using the Illumina HiSeq 4000 platform. After trimming the adaptor and removing the low-quality reads, 1,380,323,402 (98.28%) reads were retained as high-quality clean reads. These clean reads were mapped to the reference as the full-length transcriptome. The mapping ratios for the 18 cDNA libraries ranged from 73.97 to 83.78%. Using these data, the normalized expression data were calculated and normalized gene expression was analyzed by PCA (Fig. 3a). Two clusters were clearly defined by PCA, which contained the DPC group and control for each cluster. The first principal component, PC1, summarized 30.7% of the whole variability and discriminated samples according to the treatment. The second principal component, PC2, and the third principal component, PC3, summarized 25.1 and 17.4% of the whole variability and discriminated samples, respectively. The DEG analysis showed that the comparison between C2 and D2 groups had the most DEGs (a total of 6012 genes, which contained 3227 upregulated genes and 2785 downregulated genes). D1 showed more upregulated genes compared to D2 and D3 groups, while less downregulated genes were found in D1 than in D2 and D3 groups. In addition, most DEGs in C2-vs-D2, C1-vs-C2 (2895 DEGs), and D1-vs-D2 (3157 DEGs) also showed a large number of differentially expressed genes (Fig. 3b).

### Functional analyses of DEGs between C2 and D2 groups

**Fig. 4:**
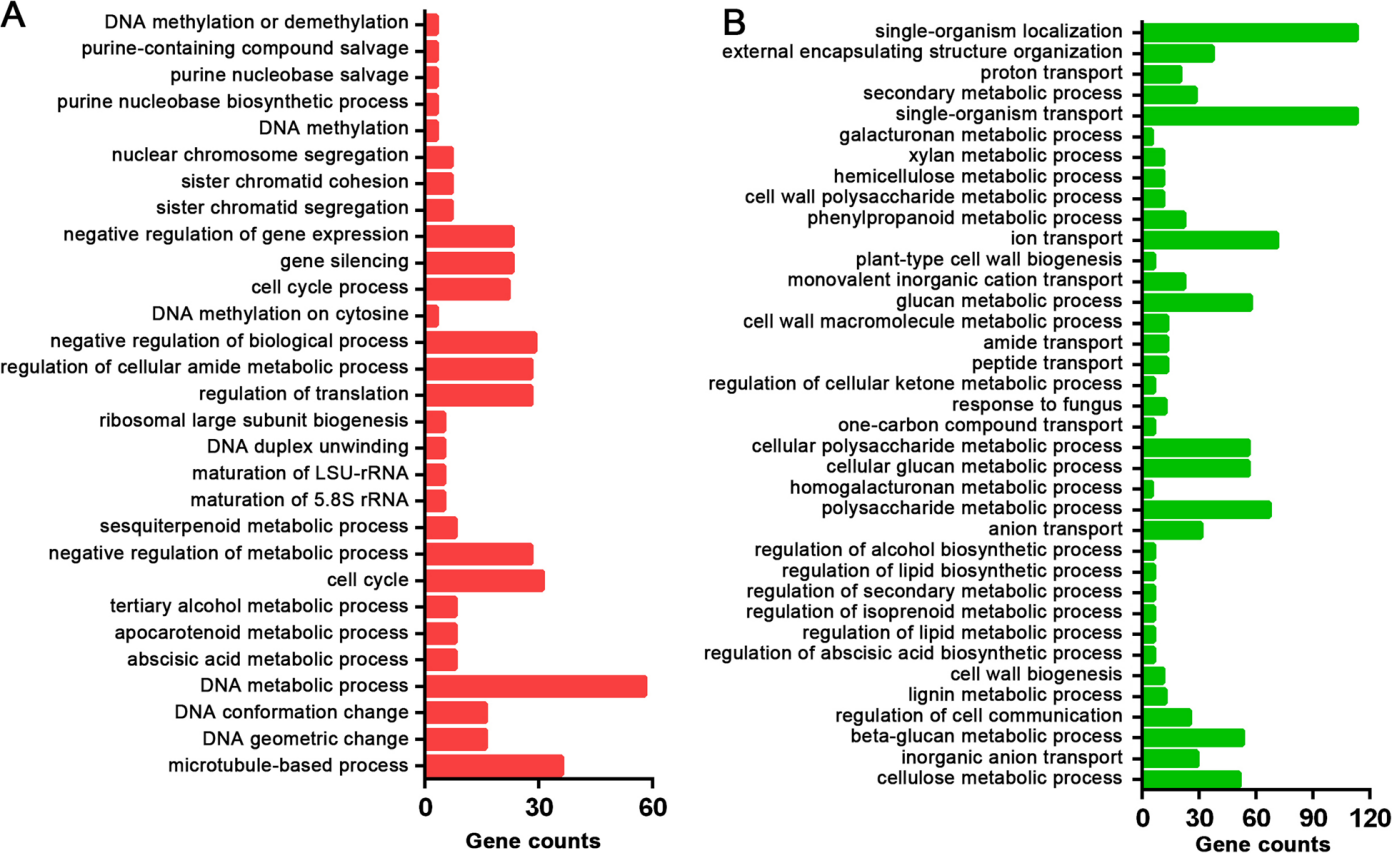
GO enrichment analysis result of upregulated genes **a** and downregulated genes **b** from C2-vs-D2 comparison

**Fig. 5:**
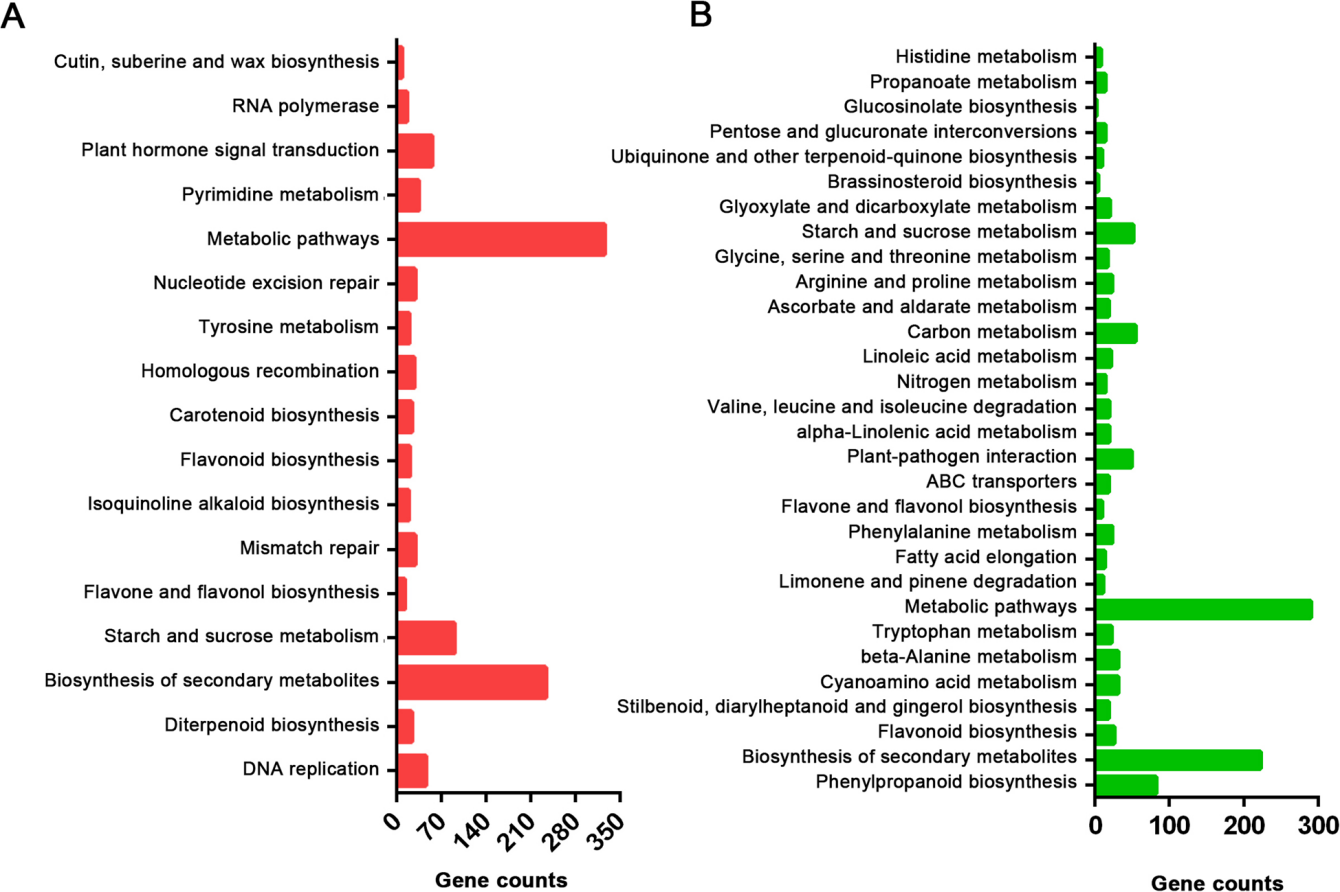
KEGG enrichment analysis result of upregulated genes **a** and downregulated genes **b** from C2-vs-D2 comparison

To illustrate the functions of the DEGs after DPC treatment, GO enrichment and KEGG enrichment analyses of the comparison of C2 and D2 with the most DEGs were performed. The upregulated and downregulated genes were annotated in 29 and 37 GO terms, respectively (Fig. 4a, b). The GO enriched terms with the four most upregulated genes were DNA metabolic process, negative regulation of biological process, regulation of translation, and regulation of cellular amide metabolic process. Meanwhile, the GO enriched terms with the two most downregulated genes were single-organism transport and single-organism localization (Additional file 4). KEGG enrichment analysis showed that 17 and 30 pathways were enriched in the upregulated and downregulated genes, respectively (Fig. 5a, b). Either for the upregulated genes or downregulated genes, metabolic pathways and biosynthesis of secondary metabolites were the top two enrichment KEGG pathways with the most genes. Among the upregulated genes, 55 were found to increase in the plant hormone signal transduction pathway. Meanwhile, phenylpropanoid biosynthesis, flavonoid biosynthesis, favone and flavonol biosynthesis, and glucosinolate biosynthesis were enriched in the downregulated genes (Additional file 5). These KEGG pathways were associated with the growth and development of internodes.

### WGCNA and hub genes

**Fig. 6:**
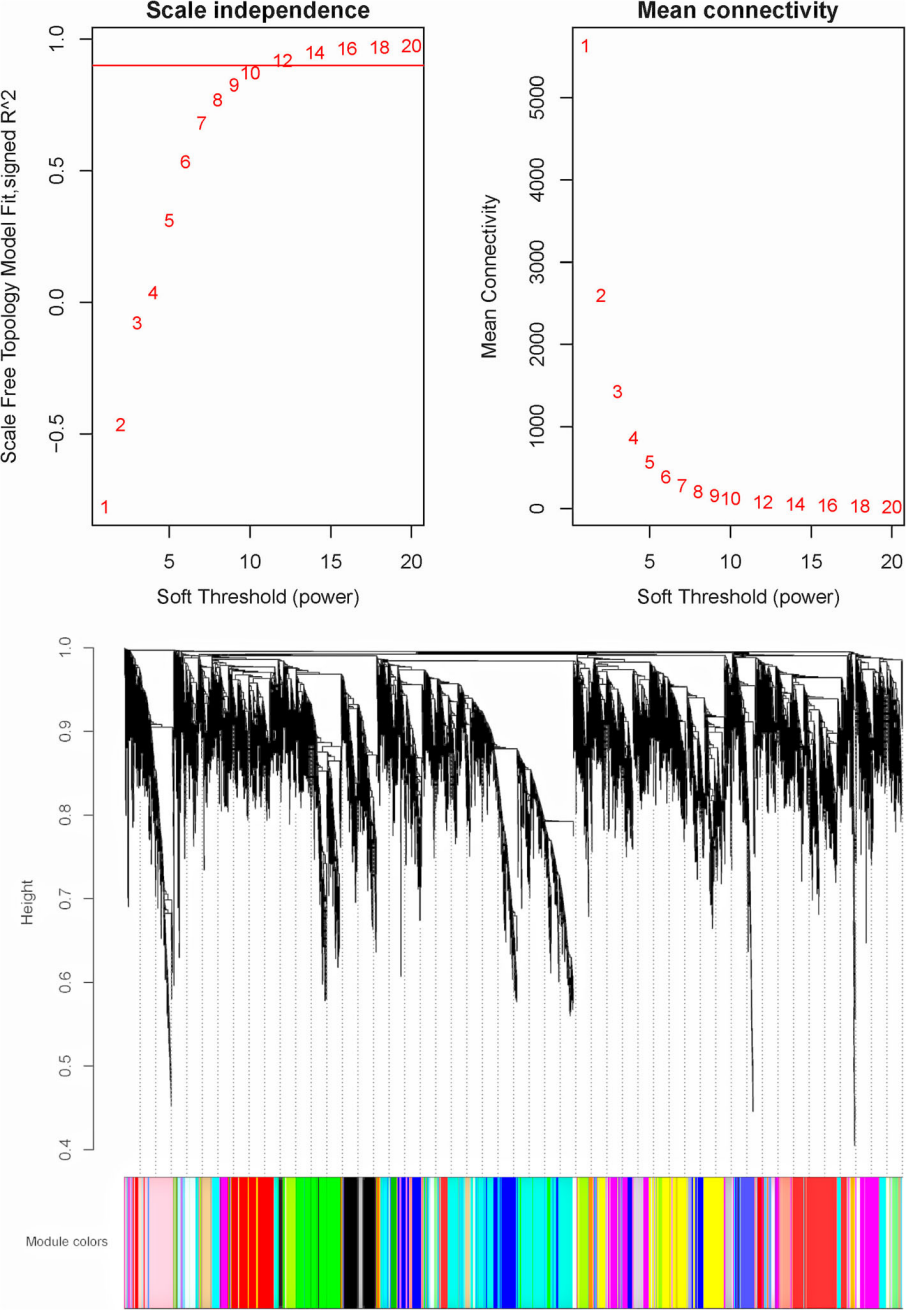
WGCNA analysis of internode transcriptomes. **a** The influence of soft-thresholding power on scale-free fit index. **b** The influence of soft-thresholding power on the mean connectivity. **c** Cluster dendrogram of the dissimilarity clustering using a consensus topological overlap. Modules were assigned different colors

**Fig. 7:**
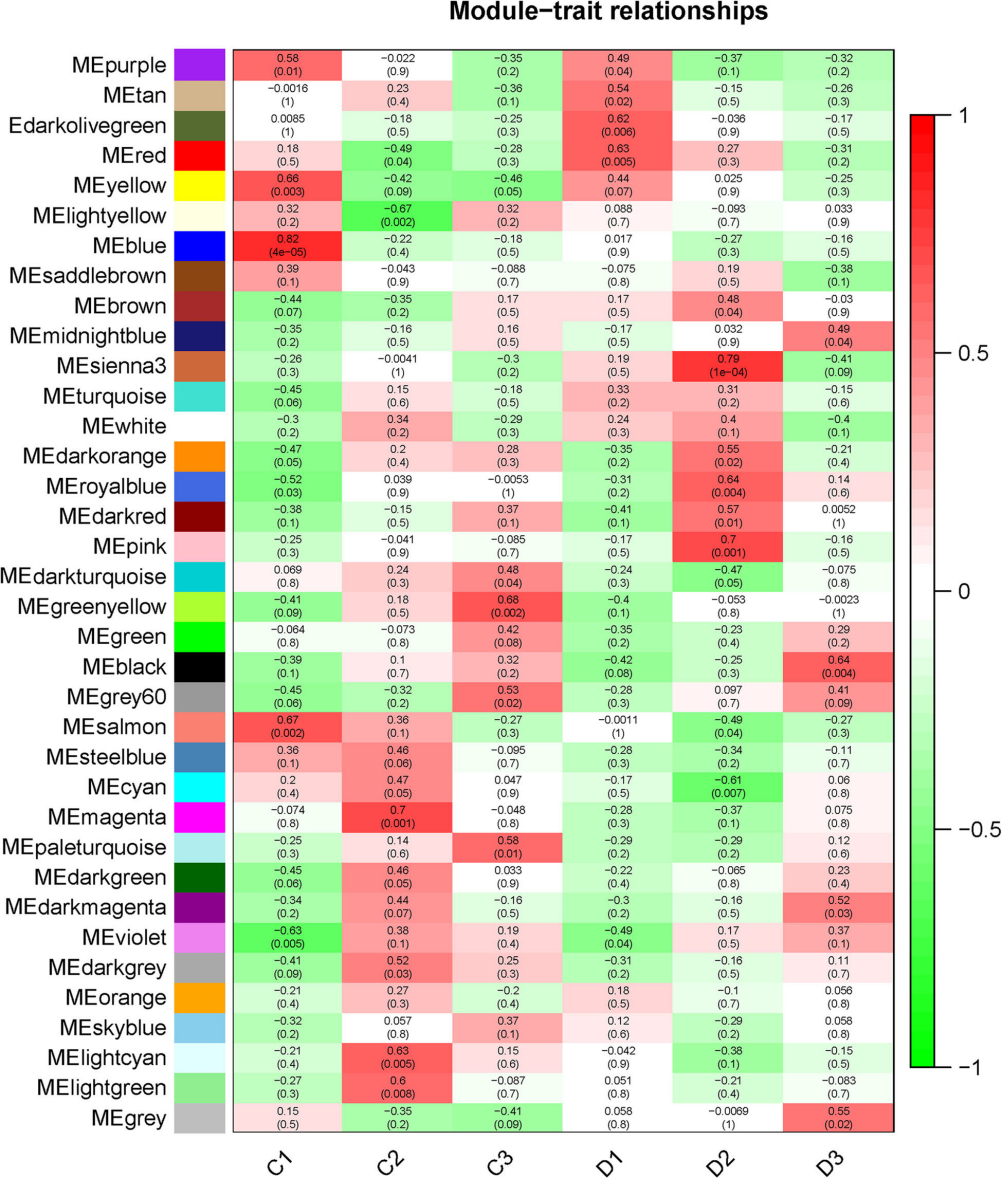
Heatmap of the module-trait relationship between different groups and gene modules. Sienna3 is the top positive module that correlated with D2 group. Values in each box represent the correlation coefficient between modules and traits. Values in brackets from each box represent the *P*-values for the correlation test

**Fig. 8:**
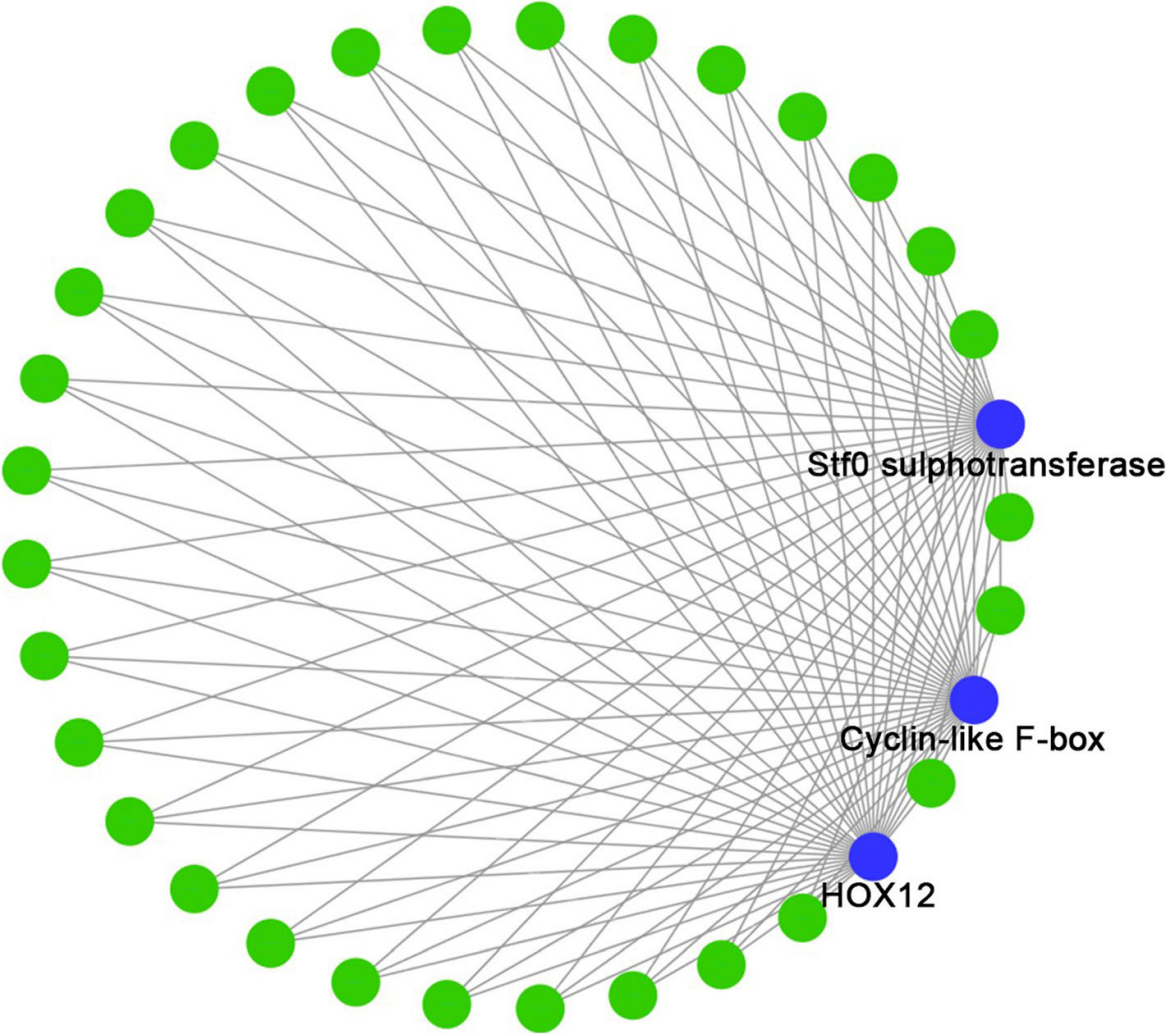
Identification of hub gene in sienna3 by Cytoscape. Blue cycles represent the hub genes while green cycles show other genes. Grey lines show the correlations between the genes

The WGCNA divided the genes into 36 modules (Fig. 6). Based on the identification of DEGs, we focused on the D2 group. This group contained significant gene expression changes, which is the crucial stage for internode elongation. We found that sienna3 was the module that most significantly correlated with the D2 stage (*p*=1e-4) (Additional file 6) (Fig. 7). The sienna3 module contained 33 genes and the top three hub genes, namely Stf0 sulfotransferase, cyclin-like F-box, and HOX12, were identified in this module. These three hub genes correlated with 30 genes (Additional file 7) (Fig. 8).

### Validation of RNA-seq result

**Fig. 9:**
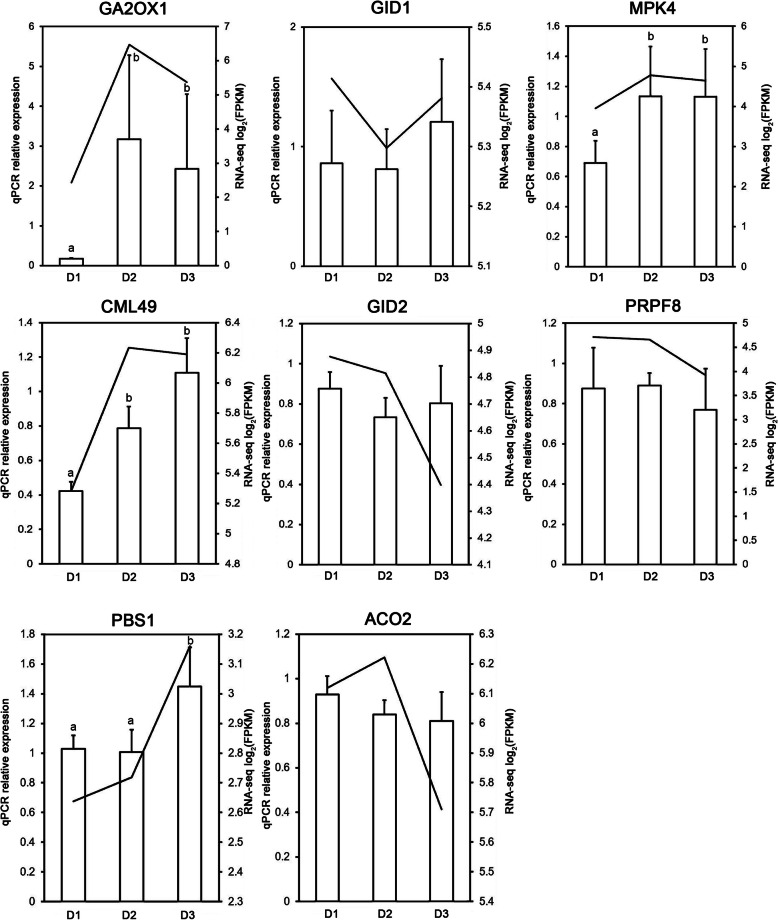
Validation of RNA-seq results by qPCR. The line charts show the log_2_(FPKM) values of the genes, and the bar charts show the relative expression from qPCR results. Different letters represent significant differences among the groups

**Fig. 10:**
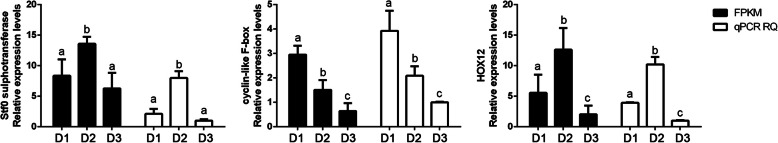
Expression of the three hub genes including Stf0 sulfotransferase **a**, cyclin-like F-box **b**, and HOX12 **c** in D1, D2, and D3 groups. The result of RNA-seq and qPCR was shown by black bars and white bars, respectively. Different letters represent significant differences among the groups

qPCR was used to validate the RNA-seq results. Randomly, nine genes were selected for the analysis. Except for *GID2* and *PBS1*, the other six tested genes, *GA2OX1*, *GID1*, *MPK4*, *CML49*, *PRPF8*, and *ACO2*, showed similar qPCR results to those of the RNA-seq. Moreover, the expression trend of six out of eight genes from qPCR and RNA-seq was highly consistent, indicating that the majority of genes had the same tendency (Fig. 9). The three hub genes, Stf0 sulfotransferase, cyclin-like F-box, and HOX12, were also analyzed by qPCR, and the results were similar between both qPCR and RNA-seq (Fig. 10). These results showed the high reliability of the RNA-Seq data.

## Discussion

Sugarcane is the main source of sugar in the industry, accounting for 79% of the sugar production worldwide. Attempts at developing techniques for controlling the growth of sugarcane, accelerating the yields, and culturing biotechnology for sugarcane resulted in varied uses of GA and DPC. These are two chemicals that regulate plant growth in sugar farming with different effects. GA stimulates sugarcane internode elongation by regulating the genes associated with zeatin biosynthesis, nitrogen metabolism, and plant hormone signal transduction pathway [41], while DPC suppresses sugarcane growth. However, compared to the clear mechanism of GA-stimulated growth, the molecular mechanisms of DPC are unclear. Thus, in the present study, we focused on the transcriptomic regulation by DPC on sugarcane and discussed the key genes that mediate its growth-suppressive effect.

First, to obtain a high-quality reference for gene annotation, we generated a full-length transcriptome from sugarcane, which was sequenced using the PacBio Sequel platform, thereby generating 72,671 isoforms. Compared to Illumina platforms, the PacBio Sequel platform could gain longer transcripts, which is an advantage in the construction of high-quality references for short sequence analysis. The present study generated reads with N50 at 3011 bp. These long reads guarantee longer contigs and isoforms for subsequent transcriptome analysis [42]. Notably, it turns out that the N50 was 3073 for the isoforms in the present study. Sugarcane is a widely cropped plant and to date, a large number of different varieties have been developed. Of these are the Guitang varieties developed from Guangxi, which have become a series of varieties planted in southern China [43]. GT42, belonging to the Guitang varieties, is a new breeding line with higher sugar productivity [43]. Although the genome of sugarcane was reported on until 2018, the genome data may differ among varieties [44]. Our study is the first to report the full-length transcriptome of GT42. It is our belief that these data would accelerate the studies on new high-yielding crops and provide a high-quality reference when analyzing the Illumina short reads. They also provided a chance to illustrate the function of internodes in GT42. Notably, the most abundant GO term regarding the biological process of GT42 isoforms, included metabolic process and cellular process. Thus, this functional isoform showed similar assignment of function to previous results from sugarcane [44–46]. Based on these data, GT42 had a functional constitution similar to that of other sugarcane varieties. The present full-length transcriptome was the first to generate general information on GT42 and provided a high-quality reference transcriptome for further investigation of this variety.

DPC is one of the most successful and widely used chemicals for regulating plant growth. Its application has been shown to reduce internode length and leaf size in cotton and sugarcane [12]. The present study also suggested that DPC inhibited internode length in GT42, which was similar to previous results. After understanding the effects of DPC on internode growth, the next question is to determine the molecular mechanism of the function of DPC in sugarcane. In doing so, we used RNA-seq to show the whole profile of gene expression regulation. Using the HiSeq technique, we obtained millions of short reads to reveal the expression in different stages induced by DPC treatment. Thanks to the high-quality full-length transcriptome data, the mapping ratios for these libraries covered 73.97 to 83.78%. The comparison between C2 and D2 had the most DEGs, which was 6012 genes. This number of DEGs was much higher than that in C1-vs-D1 and C3-vs-D3, suggesting that the gene expression changes between the control and DPC treatment were mainly in the second stages; namely, after six days post application via spraying. In a study on cotton spraying with DPC, the 96 h post spraying significantly had the most DEGs compared to the 48 h and 72 h stages. From this, it seems that DPC resulted in changes in gene expression over the long-term course of four to six days. Gene expression regulation by DPC is not an acute effect. After 10 days, the effects of DPC on gene expression were diminished. We supposed that the most effective period of DPC-regulated gene expression was six days.

The KEGG enrichment analysis showed that the expression levels of 55 genes in the plant hormone signal transduction pathway had increased from DPC treatment. Internode growth is controlled by several hormonal genes, such as G biosynthesis genes, auxin-related genes, and ethylene genes. It has been reported that GA treatment can significantly upregulate these genes, while DPC may suppress hormone expression. Specifically, in *Agapanthus praecox*, auxin-related genes were shown to be inhibited by DPC treatment [47]. Surprisingly, the present study also indicated that DPC increased the expression levels of several hormonal genes. This difference may be due to the different species examined. Therefore, sugarcane may have a different response to DPC at the molecular level. We also found that several key pathways could be downregulated by DPC, such as phenylpropanoid biosynthesis, flavonoid biosynthesis, favone and flavonol biosynthesis, and glucosinolate biosynthesis, which were enriched. The phenylpropanoid pathway provides metabolites for plant growth, which contributes to the requirement of lignin biosynthesis [48]. Moreover, favone, flavonol, and glucosinolate are key metabolites for internode growth [49, 50]. Flavonol biosynthesis could be affected by light intensity and, in previous studies, led to different growth appearances in Ginkgo (*Ginkgo biloba*) [51]. Meanwhile, the glucosinolate concentration, influenced by sulfur and nitrogen supplementation, was associated with the growth of broccoli [52]. The downregulation of genes in these pathways may lead to the shortening effects of sugarcane internodes.

To determine the key gene modules and hub genes from the effects of DPC treatment, WGCNA was performed. In this sienna3, 33 genes were found highly correlated with the three hub genes. Therefore, the most critical genes play a key role in the module. Hub genes are the genes that correlate with other genes in expression levels, which could be identified by mathematical methods. The top three identified in this study were Stf0 sulfotransferase, cyclin-like F-box, and HOX12. Stf0 belongs to the sulfotransferase family, which affects root development processes, elongation growth, and gravitropism [53]. In several plants, including *Medicago truncatula*, *Lotus japonicus*, and *Arabidopsis thaliana*, cyclin-like F-box genes were expressed in all the tissues containing highly active dividing cells. Knockdown of this gene resulted in the accumulation of CYCB1:1, suggesting that the cyclin-like F-box gene could regulate the cell cycle in dividing cells [54]. Furthermore, it has been reported that HOX12 regulates panicle exsertion via modulating *EUI1* gene expression [55]. These three hub genes were correlated with the other genes in the sienna3 modules. Based on this information, it could be concluded that Stf0 sulfotransferase, cyclin-like F-box, and HOX12 mediated a gene group and constituted a gene network that contributed to the DPC-induced effects on sugarcane growth.

## Conclusion

In summary, the full-length GT42 transcriptome was first reported in this study, thereby providing an informative resource for sugarcane breeding and transcriptome analysis. RNA-seq suggested that the main effects of DPC on sugarcane gene expression occurred six days post spraying. Furthermore, the significantly enriched gene function categories contained several pathways related to internode growth, including multiple pathways that participated in the production of metabolic products. Additionally, the gene modules included 33 genes that were highly correlated with the stage of six days post spraying in the DPC group, showing a potential role in the response to DPC. Among these genes, Stf0 sulfotransferase, cyclin-like F-box, and HOX12 were hub genes that may regulate all the other genes in this module. Further studies should focus on determining the function of these key genes in detail, especially with regards to controlling internode growth affected by DPC.

## Methods

### Sugarcane preparation

All the sugarcane samples used were bred at the Sugarcane Research Institute (SRI), Guangxi Academy of Agricultural Sciences in Nanning, China. The sugarcane variety, GT42, was sourced from the SRI Experimental Farm in Nanning, China. The team selected 10-month-old cane stalks to obtain buds in the middle internodes, which were then cut into setts from a single bud. The setts were incubated at 52 °C for 30 min to eliminate pathogenic bacteria and subsequently were planted in a moist sandbox and maintained in an artificial climate box (Essenscien, USA). The culturing conditions were as follows: temperature 28.0±0.1 °C, humidity: 75±1.5% RH, photoperiod 12 h light and 12 h dark with 100% full light (light intensity 25,000 lx). Once the seedlings grew their first two leaves, they were transferred to plastic pots (35 cm width × 35 cm length × 50 cm height); in each pot, two seedlings were planted. After five days, the seedlings were randomly divided into two replicates. The seedlings were cultivated to the pre-elongation stage, which contained 9–10 leaves, defined as the early elongation stage. In this stage, the DPC group was sprayed with 200 mg/L DPC (Solarbio Life Science, Beijing, China) until the solution began to drip from the leaves. Water was sprayed on the control group in a similar pattern. All the sugarcane pots were placed in a greenhouse in 18 rows with 1.2 m width. The first three columns belonged to the control group and the last three columns belonged to the DPC group. At 3, 6, and 12 days post spraying, the third internodes were collected for further assays. Control samples from 3, 6, and 12 days post spraying were named C1, C2, and C3, respectively. Similarly, the samples of the DPC group from 3, 6, and 12 days post spraying were named D1, D2, and D3, respectively. All samples were stored at − 80 °C until RNA isolation. For each group, at different time points, three biological replicates were collected for analyses.

### Determination of growth performance

Sugarcane growth performance was measured in the control and DPC groups. At 3, 6, and 12 days post spraying, we measured the stalk height from the soil surface to the dewlap of the youngest fully expanded leaf, as well as the length of the internodes. For each group, five plants were randomly chosen for measurement. The whole height and the first seven internode lengths (from the shoot apex of 10 matured plants) were measured as well.

### PacBio Iso-Seq

To obtain an accurate reference for the genes in sugarcane, full-length transcriptome sequencing was performed. RNA libraries of internodes from one mature sugarcane at 10 months of age were prepared. The mRNAs were first enriched with oligo (dT) magnetic beads, and the full-length cDNAs were synthesized using Clontech SMARTer PCR cDNA Synthesis Kit (Pacific Biosciences, USA). From this, three libraries with different lengths (1–2 kb, 2–3 kb, and 3–6 kb) were constructed. Sequencing was performed on a PacBio Sequel System (Pacific Biosciences, USA) and the raw sequences were analyzed using SMRT Link v5.0.1 software. Based on the primer at 5′ and 3′ as well as ploy-A; the full-length, non-full-length, chimeric, and non-chimeric categories were identified. The non-full-length sequences were polished using the Quiver algorithm, while the Illumina RNA-seq data were used to correct the low-quality sequences. The sequences were annotated using the nr, SwissProt, COG/KOG, GO, and KEGG pathways, and the unannotated sequences were further used for CDS prediction.

### Preparation of RNA-seq libraries

Total RNA from three plants in each group was isolated using RNA Trizol (Invitrogen, Carlsbad, CA, USA) following the manufacturer’s instructions. A total of six RNA-seq libraries (three from the control group and three from the DPC group) were prepared for next-generation sequencing. The quantity and integrity of the total RNA was assayed using an Agilent 2100 bioanalyzer (Agilent, Santa Clara, CA, USA). The mRNAs were enriched by oligo (dT) magnetic beads and fragmented using fragmentation buffer. First-strand cDNA was synthesized using random primers 6-bases long. The second-strand cDNA was then synthesized using DNA polymerase I. Subsequently, the cDNAs were isolated using a QIAquick PCR extraction kit (Qiagen, Hilden, Germany), and ligated with Illumina sequencing adapters. Finally, the cDNAs were purified using agarose gel electrophoresis and amplified by PCR to generate RNA-seq libraries. The RNA-seq was performed on an Illumina HiSeq™ 4000 by Gene Denovo Biotechnology Co. (Guangzhou, China).

### Transcriptome mapping and differentially expressed gene (DEG) identification

The sequencing adaptor was first trimmed, then low-quality reads with unknown nucleotides (N) ratio > 10% or Q-value ≤20 were removed. The retained reads were high-quality clean reads that were used for the following analyses. The clean reads were mapped to the reference transcriptome sequence using the full-length transcriptome by TOPHAT (version 2.0.9) [56], and the relative gene expression was calculated and normalized by Fragments Per Kilobase of transcript per Million mapped reads (FPKM). Furthermore, principal component analysis (PCA) was performed using the R package (http://www.r-project.org/) to evaluate the reproducibility of the biological replicates. When the genes with a false discovery rate (FDR) < 0.05 and log_2_(fold change) > 1 or <− 1 were compared between the control group and DPC group, the genes were identified as DEGs.

### Functional annotation of DEGs

To define the function of DEGs, enrichment analysis of Gene Ontology (GO) and Kyoto Encyclopedia of Genes and Genomes (KEGG) pathways were performed. The DAVID online tools (http://david.ncifcrf.gov/) were employed for the enrichment analysis. The GO items with adjusted *P*≤0.001 and KEGG pathways with *P*≤0.001 were considered to be significantly enriched.

### WGCNA

The WGCNA was performed to identify key gene groups and hub genes based on the FPKM using the R package [57]. The data were first filtered according to the 25% variation based on variance (Standard Deviation/Mean) across samples. Further, the FPKM matrix of the retained genes was used to create a weighted adjacency matrix. The soft threshold power (β) set at 10, was selected to perform scale-free topology. The parameters for construction of the gene module were power = 8, minimum module size = 30, and branch merge cut height = 0.25. The correlations between gene modules and treatment groups were evaluated using correlation coefficients. The D2 group was the most notable treatment group owing to the large number of DEGs identified and we chose the top-correlated module (sienna3) of the D2 group for further analysis. First, GO and KEGG pathway enrichment analyses of the module were performed using KOBAS v3.0 (http://kobas.cbi.pku.edu.cn/). The top three hub genes were identified using Cytohubba (http://apps.cytoscape.org/apps/cytohubba) and the network was plotted using Cytoscape v3.7.1.

### Real-time quantitative PCR analysis of genes (qPCR)

Total RNA from internode tissues in the control and DPC groups were isolated and tested as described in section 2.1. The first-strain cDNAs were synthesized using the PrimeScript RT Reagent Kit with gDNA Eraser (Takara, Japan). Primers used for qPCR were designed by Primer Premier 5.0 (Applied Biosystems, Waltham, MA, USA) according to the gene sequences from the PacBio Iso-Seq (Additional file 1). The qPCR was performed on an Analytik Jena qTOWER 2.2 fluorescence quantitative PCR instrument (Germany). Furthermore, the PCR program used was as follows: 95 °C for 5 min for initial denaturation, followed by 45 amplification cycles at 95 °C for 10 s and 60 °C for 20 s. Five biological replicates were tested for the amplification of each sample and *EF1A* was used as the control gene. To confirm the specificity of the PCR reaction, a melt curve analysis was performed. Additionally, the relative expression of the genes was calculated by the 2^-ΔΔCT^ method.

### Data statistical analysis

The growth rate, sugarcane height, internode length, and relative expression are presented as mean ± standard deviation (SD). The significant differences among the groups were determined using one-way analysis of variance (ANOVA) and post hoc Tukey’s test using SPSS statistical software package (v19.0, SPSS, Chicago, IL, USA). *P* values < 0.05 were considered to be significantly different.

## Supplementary Information

**Additional file 1.** The primers used in this study.

**Additional file 2.** Summary of full-length transcriptome of sugarcane.

**Additional file 3.** Annotation results of isoforms from full-length transcriptome of sugarcane.

**Additional file 4.** GO enrichment analysis of DEGs.

**Additional file 5.** KEGG enrichment analysis of DEGs.

**Additional file 6.** Correlations between modules and the groups. (CSV 4 kb)

**Additional file 7.** Gene networks of the three hub genes including Stf0 sulfotransferase, cyclin-like F-box, and HOX12. (CSV 2 kb)

## Data Availability

Data for the sugarcane used in the RNA-Seq analysis are accessible at NCBI under the BioProject accession number PRJNA633918.

## Abbreviations

DPC: Mepiquat chloride
WGCNA: Weighted gene co-expression network analysis
GA: Gibberellin
CTK: Cytokinins
ABA: Abscisic acid
ETH: Ethyne
BR: Brassinosteroid
GA20-OX1: GA20-oxidase
GID1: Gibberellin receptor
SRI: Sugarcane Research Institute
GO: Gene Ontology
KEGG: Kyoto Encyclopedia of Genes and Genomes
FPKM: Fragments Per Kilobase of transcript per Million mapped reads
CCS: Circular-consensus sequence
FLNC: Full-length non-chimeric
PCA: Principal component analysis
SD: Standard deviations
ANOVA: One-way analysis of variance
DEGs: Differentially expressed genes
